# Early Detection and Curative Endoscopic Submucosal Dissection of a 4-mm Signet Ring Cell Microcarcinoma in a Reconstructed Gastric Tube: A Case Report

**DOI:** 10.7759/cureus.100099

**Published:** 2025-12-25

**Authors:** Masao Sato, So Takahashi, Masahide Shimura, Naohiro Kobayashi, Suguru Arata, Takahiro Domen, Misa Yamauchi, Katsunori Iijima

**Affiliations:** 1 Department of Gastroenterology, Yuri Kumiai General Hospital, Yurihonjo, JPN; 2 Department of Pathology, Yuri Kumiai General Hospital, Yurihonjo, JPN; 3 Department of Gastroenterology and Neurology, Akita University Graduate School of Medicine, Akita, JPN

**Keywords:** egd, endoscopic submucosal dissection, esd, esophagogastroduodenoscopy, gastric cancer, gastric tube, gastric tube cancer, reconstructive gastric tube, signet-ring cell carcinoma, undifferentiated gastric cancer

## Abstract

Gastric tube cancers are rare but clinically challenging entities that may arise years after esophageal or gastric surgery. Due to anatomical alteration and subtle mucosal changes, early detection remains difficult, particularly for signet-ring cell carcinoma (SRCC), which often progresses unnoticed. We report a rare case of micro SRCC arising in a reconstructed gastric tube, which was detected early and curatively resected by endoscopic submucosal dissection (ESD). The patient was initially diagnosed with adenocarcinoma of the esophagogastric junction during a routine esophagogastroduodenoscopy (EGD) performed as part of a medical check-up at a local clinic. He underwent ESD, but the pathological evaluation revealed a positive vertical margin, and he was subsequently referred to a tertiary center. Curative resection was achieved through open proximal gastrectomy combined with robot-assisted thoracoscopic esophagectomy of the middle and lower esophagus. Two years postoperatively, a follow-up EGD revealed a small, flat, whitish lesion measuring 5 mm on the lesser curvature of the gastric angle in the reconstructed gastric tube. Biopsy results revealed undifferentiated-type gastric cancer, predominantly composed of SRCC. Given the lesion’s clearly shallow invasion and very small size on endoscopic examination, ESD was performed. Pathological findings confirmed a 4 × 4 mm SRCC limited to the mucosal layer, with negative horizontal and vertical margins, and no lymphovascular invasion, consistent with curative resection. This case highlights the importance of vigilant long-term endoscopic follow-up after upper gastrointestinal surgery and demonstrates that even micro SRCCs in reconstructed gastric tubes can be successfully detected and treated at an early stage by ESD.

## Introduction

Following esophageal surgery, cancer may develop in the reconstructed stomach, a condition known as gastric tube cancer (GTC). GTC is a rare but increasingly recognized long-term complication after esophagectomy, with reported incidences ranging from 0.2% to 5.1% [1]. A multicenter study also reported that 60% of GTC cases occurred in the anal third of the gastric tube, 79.7% were histologically classified as differentiated adenocarcinomas, and the median interval from esophagectomy to GTC diagnosis was six years, with approximately 25% of cases diagnosed more than 10 years postoperatively [2]. In addition, signet-ring cell carcinoma (SRCC) is a subtype of undifferentiated gastric cancer. Micro SRCC lesions detected at an early, curable stage remain exceedingly rare, with limited cases reported in the literature. While the prognosis of gastric SRCC in its early stages is reportedly comparable to that of non-signet-ring cell types, the prognosis becomes significantly worse once the disease progresses [3]. One factor contributing to this poor prognosis is the difficulty in early detection, as early-stage SRCCs often present with a flat or depressed morphology, making endoscopic recognition challenging [4]. As a result, diagnosis may be delayed until the disease has advanced to scirrhous gastric cancer, which is associated with a markedly poor prognosis [5,6]. Here, we report a rare case of a 4-mm micro SRCC detected during postoperative surveillance esophagogastroduodenoscopy (EGD) in a reconstructed gastric tube following open proximal gastrectomy and robot-assisted thoracoscopic resection of the middle and lower esophagus. The lesion was curatively resected by endoscopic submucosal dissection (ESD). We present this case with a review of the relevant literature.

## Case presentation

A 69-year-old man presented for postoperative surveillance. He had no current complaints. His past medical history included esophagogastric junction cancer at age 68. His chronic medications included vonoprazan fumarate 20 mg/day, mosapride citrate hydrate 15 mg/day, pancreatic enzyme granules 1.5 g/day, and rikkunshito, a traditional Japanese Kampo medicine, 2.5 g/day. He had no known drug allergies. His family history was notable for his father’s death from laryngeal cancer at age 75. Socially, the patient did not consume alcohol, had smoked 30 cigarettes per day from age 20 to 67, and was previously employed as a construction worker. At age 59, a urea breath test for *Helicobacter* *pylori *(*H. pylori*) conducted at a local clinic was negative.

**Figure 1:**
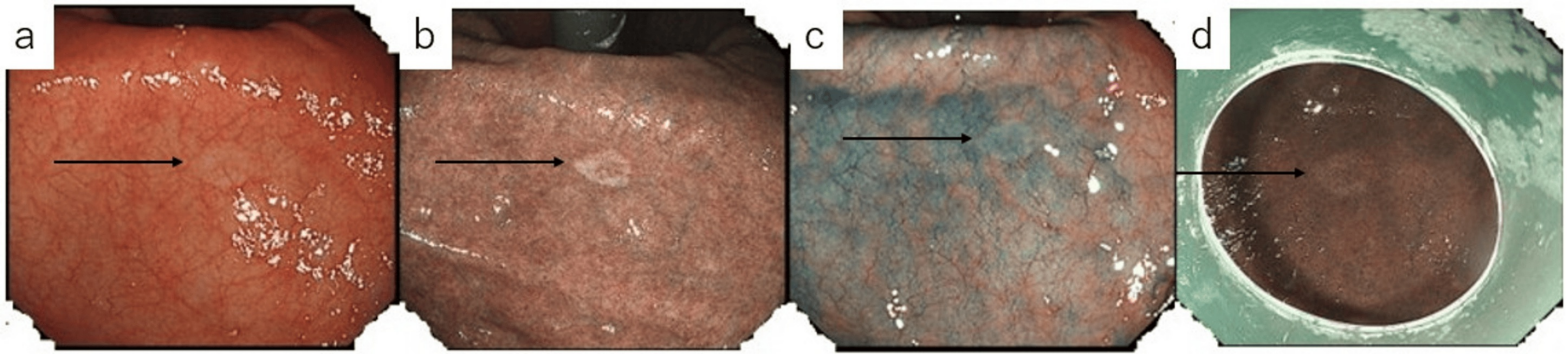
Endoscopic findings at the time of lesion detection and at the initiation of endoscopic submucosal dissection (ESD). The lesion is indicated by black arrows. (a) White‑light endoscopy at initial detection showing a subtle whitish lesion. (b) Narrow band imaging (NBI) at initial detection, providing clearer visualization compared with white‑light observation. (c) White‑light endoscopy after indigo carmine dye spraying; the lesion shows minimal surface irregularity, and visibility remains largely unchanged. (d) NBI at the beginning of ESD demonstrating preserved visibility of the lesion.

In April 2023, during a routine medical checkup, an EGD performed at a local clinic revealed a lesion at the esophagogastric junction. ESD was performed in July, and pathological examination diagnosed moderately differentiated adenocarcinoma (tub2). Due to a positive vertical margin, the patient was referred to a tertiary center for surgical management. In August, he underwent an open proximal gastrectomy and robot-assisted thoracoscopic resection of the middle to lower esophagus, achieving curative resection. Follow-up contrast-enhanced computed tomography (CT) in October 2024 showed no evidence of recurrence. In January 2025, a surveillance EGD revealed no abnormalities at the anastomotic site or elsewhere, including the lesser curvature of the gastric angle. However, in June 2025, he began experiencing reflux symptoms occurring each morning. Vonoprazan fumarate 20 mg/day was initiated, with rapid symptomatic relief. A follow-up EGD later that month showed no anastomotic abnormalities, but a 5-mm flat, whitish, and discolored area was observed on the lesser curvature of the gastric angle in the reconstructed gastric tube, which had a non-atrophic background mucosa (Figure 1). Targeted biopsy using biopsy forceps (Baby GI Jaw®, Medical Leaders Co., Ltd., Tokyo, Japan) revealed group 5 undifferentiated adenocarcinoma, predominantly SRCC (sig > por). Histological evaluation for *H. pylori* was negative.

Upon receiving the results at our outpatient clinic, the patient demonstrated psychological distress. We provided detailed explanations about the lesion and engaged in shared decision-making. Given the lesion’s extremely small size and clearly shallow invasion on endoscopic inspection, ESD was proposed. A contrast-enhanced CT scan was performed for depth assessment. The lesion could not be clearly visualized, and there was no evidence of perigastric lymphadenopathy or distant metastases. In July, the patient was admitted to our gastroenterology department for ESD of the gastric tube lesion.

At the time of initial presentation to our department, the patient's vital signs and physical examination were unremarkable, and he reported no subjective symptoms. The patient had no subjective symptoms. Laboratory findings at the time of initial evaluation are summarized in Table 1.

**Table 1:** Laboratory findings at initial presentation to our department. The mildly decreased Hb level was considered to be within the range of age-related physiological changes. The mildly elevated CRP level was considered to be within the range of physiological responses, given that less than two years had elapsed since major surgery involving gastric tube reconstruction and considering the frequency and severity of acid reflux in the reconstructed gastric tube. The tumor marker CEA was only slightly above the normal range, which was not inconsistent with the presence of early-stage gastric cancer.

Category	Parameter (Abbreviation)	Result	Reference Range	Unit
Hematology	White Blood Cell (WBC)	7200	3300-8600	/µL
	Red Blood Cell (RBC)	403×10^4^	410-530×10^4^	/µL
	Hemoglobin (Hb)	12.9	13.5-17.6	g/dL
	Hematocrit (Ht)	39.1	40-50	%
	Platelets (Plt)	25.6×10^4^	13.0-36.9×10^4^	/µL
Coagulation	Prothrombin Time-International Normalized Ratio (PT-INR)	0.95	0.85-1.15	-
	Activated Partial Thromboplastin Time (APTT)	34.7	25-40	sec
	D-dimer	0.7	<1.0	μg/mL
Biochemistry	Total Protein (TP)	6.1	6.5-8.0	g/dL
	Albumin (Alb)	3.9	4.0-5.0	g/dL
	Total Bilirubin (T-Bil)	0.38	0.2-1.2	mg/dL
	Aspartate Aminotransferase (AST)	19	13-30	IU/L
	Alanine Aminotransferase (ALT)	24	10-42	IU/L
	Alkaline Phosphatase (ALP)	63	38-113	IU/L
	γ-GTP (γ-Glutamyl Transpeptidase)	55	10-47	IU/L
	Lactate Dehydrogenase (LDH)	146	120-245	IU/L
	Creatine Kinase (CK)	53	50-170	U/L
	Amylase (AMY)	70	40-130	U/L
	Blood Urea Nitrogen (BUN)	16	8-20	mg/dL
	Creatinine (Cre)	0.74	0.6-1.1	mg/dL
	Sodium (Na)	140	138-145	mEq/L
	Potassium (K)	4.4	3.6-4.8	mEq/L
	Chloride (Cl)	103	101-108	mEq/L
	C-reactive Protein (CRP)	1	<0.3	mg/dL
	Glucose (Glu)	100	70-109	mg/dL
	Hemoglobin A1c (HbA1c)	5.9	4.6-6.2	%
Tumor Marker	Carcinoembryonic Antigen (CEA)	5.05	<5.0	ng/mL
	Carbohydrate Antigen 19-9 (CA19-9)	29.4	<37	U/mL

Clinical course after admission

**Figure 2:**
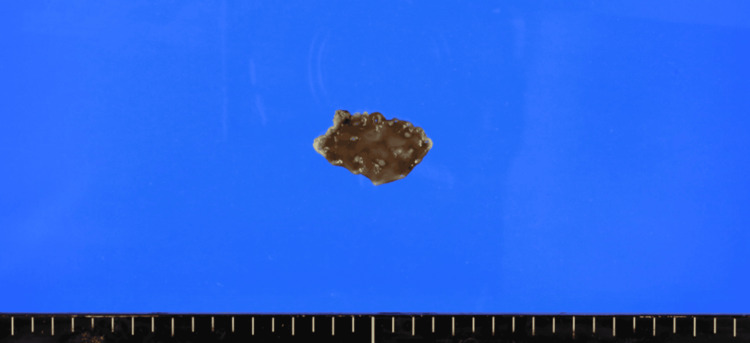
Image of the resected specimen.

ESD was performed for the GTC lesion. At the beginning of the procedure, the lesion remained clearly visible, comparable to its appearance at the time of initial detection (Figure 1). ESD was performed, and a video endoscope, GIF-H290T® (Olympus Corporation, Tokyo, Japan), was used. For submucosal injection, a disposable injection needle (Needle Master®; Olympus Corporation) was utilized. The injection solution consisted of a mixture of saline (Otsuka Normal Saline®; Otsuka Pharmaceutical Factory, Inc., Tokushima, Japan), indigocarmine (Indigocarmine®; Alfresa Pharma Corporation, Osaka, Japan), concentrated glycerin fructose sodium chloride (Hisiceol®; Nipro Corporation, Osaka, Japan), and purified sodium hyaluronate (MucoUp®; Boston Scientific Japan, Tokyo, Japan). For mucosal incision and submucosal dissection, we used a tip-type knife (Endosaber®; tip length 2.5 mm; SB-Kawasumi Co., Ltd., Kanagawa, Japan) and a disposable high-frequency knife (ITknife 2®; Olympus Corporation). For hemostasis and prophylactic coagulation of visible vessels, a disposable high-frequency hemostatic forceps (Coagrasper®; Olympus Corporation) was employed. The high-frequency generator used was VIO3® (AMCO Inc., Tokyo, Japan). During the procedure, wide marking was placed around the lesion, followed by a circumferential incision and en bloc resection with adequate safety margins to ensure both horizontal and vertical margin negativity. The resected specimen measured 30 × 17 mm (Figure 2).

**Figure 3:**
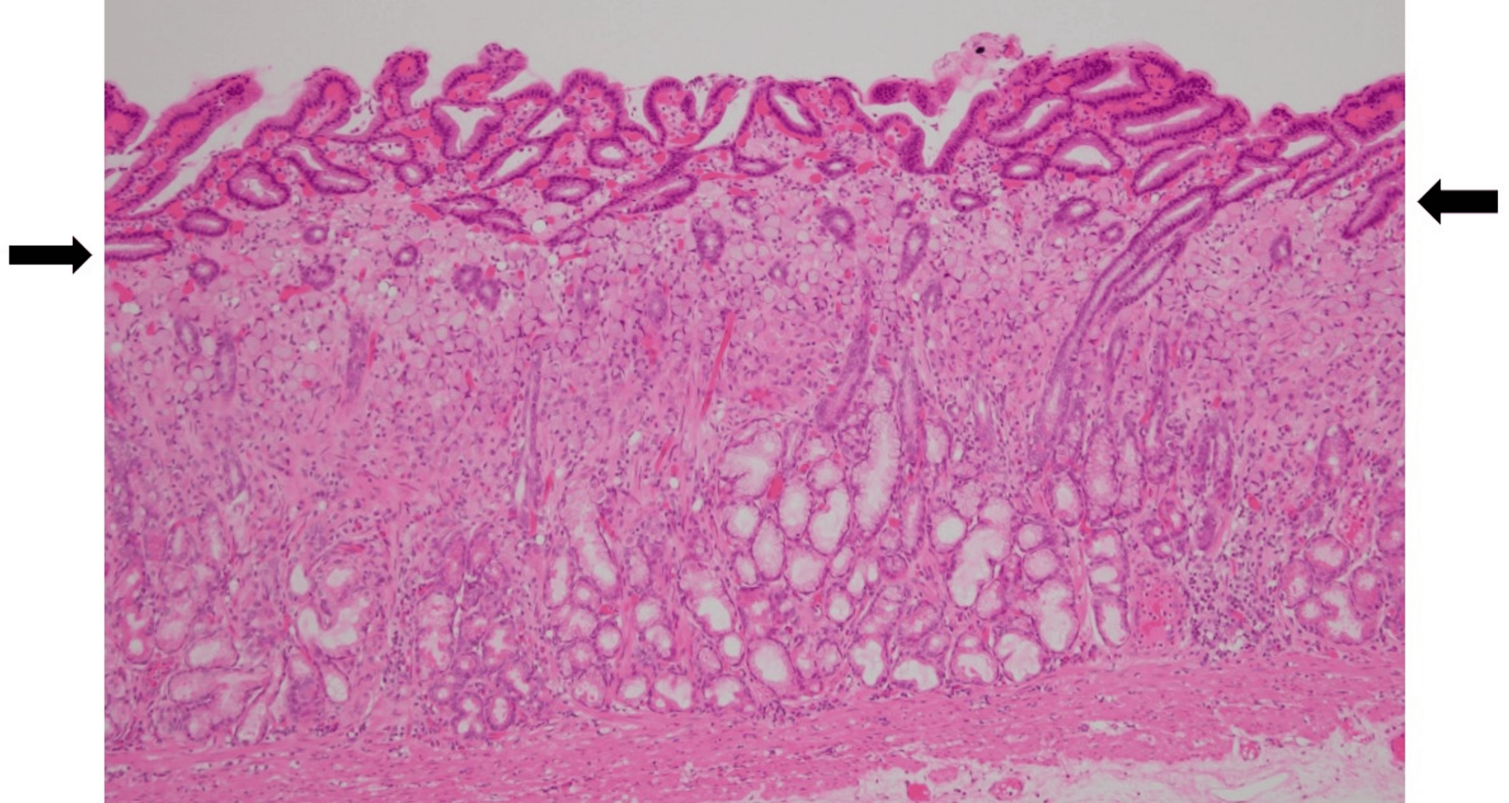
Low-power view of the pathological specimen. The superficial layer above the level indicated by the black arrows is covered by non-neoplastic mucosa.

**Figure 4:**
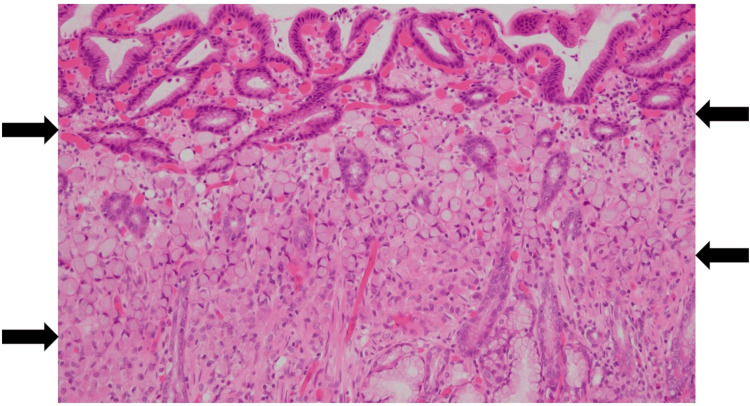
High-power view of the pathological specimen. Signet-ring cells are observed within the layer indicated by the black arrows.

Histopathological analysis revealed that the lesion was superficially covered with non-neoplastic mucosa but consisted of a 4 x 4 mm SRCC confined to the mucosal epithelium (Figures 3-4). Both horizontal and vertical resection margins were negative, and no evidence of lymphovascular invasion was identified, consistent with curative resection. The postoperative course was uneventful, and the patient was discharged on postoperative day 7.

Following discharge, the patient continued taking vonoprazan fumarate 20 mg, which had been prescribed prior to admission. A follow-up EGD performed eight weeks after the ESD confirmed scar formation at the resection site. At that time, gastric juice was collected, and a polymerase chain reaction (PCR) test using the *H. pylori *nucleic acid detection kit (Smart Gene®; Mizuho Medy Co., Ltd., Saga, Japan) returned negative. Annual surveillance EGD has been recommended, and the patient has since been discharged from our department’s follow-up. As of December 2025, no apparent postoperative complications have been observed.

## Discussion

This case report describes a rare instance of an SRCC arising in a reconstructed gastric tube that was detected at a very early stage (4 × 4 mm) and successfully resected curatively via ESD. To our knowledge, only a limited number of micro SRCC cases (<5 mm) in reconstructed gastric tubes have been reported, and few were treated endoscopically. SRCC is generally considered difficult to detect in its early phase and is known for its poor prognosis when advanced [3-6]. As such, this case offers valuable insight into the natural history of SRCC and provides important implications for routine endoscopic surveillance.

In Japan, gastric cancer is histologically classified into differentiated and undifferentiated types [4,7], whereas in Western countries, it is categorized as intestinal or diffuse type [8]. Undifferentiated types include poorly differentiated adenocarcinoma (por), SRCC (sig), and mucinous adenocarcinoma (muc) [7].

SRCC typically arises near the glandular neck region of the fundic mucosa without atrophy. It consists of signet-ring cells that accumulate intracellular mucin and lack a glandular structure [9]. In its earliest stage, the tumor remains localized to the glandular neck and endoscopically appears as a flat, discolored 0-IIb lesion. As tumor cells proliferate and involve nearly the entire mucosal layer, the lesion evolves into a depressed, discolored 0-IIc type [10]. Histopathologically, SRCC is densely distributed in the glandular neck zone, corresponding to the boundary between foveolar epithelium and fundic glands. The surface epithelium appears morphologically indistinct from the surrounding mucosa.

Because SRCC frequently occurs at the junction of the fundic and pyloric glands, careful observation of the entire circumference of the gastric angle is essential for efficient detection. In particular, the lesser curvature and posterior wall are areas prone to blind spots, necessitating meticulous endoscopic inspection. On white-light imaging, contrast between the lesion and non-atrophic background mucosa aids diagnosis. However, due to the lesion’s lack of elevation or depression, dye-based methods such as indigo carmine spraying may obscure color differences and are often unnecessary. Image-enhanced modalities such as narrow-band imaging (NBI) and blue laser imaging (BLI) have been reported to enhance contrast between SRCC and the surrounding mucosa. Additionally, biopsy of small lesions may induce local inflammation, edema, and epithelial regeneration, leading to temporary obscuration of the lesion. Thus, it is recommended to perform minimal biopsies at the lesion margins with caution [11].

Several multicenter studies, systematic reviews, and meta-analyses examining the safety and efficacy of ESD in the remnant stomach and reconstructed gastric tube have reported that ESD for early neoplastic lesions arising after surgical resection achieves comparable en bloc and curative resection rates to those in anatomically normal stomachs. Although technically challenging, the rates of adverse events such as perforation and bleeding were generally similar to those observed in standard gastric ESD procedures [12-14].

In recent years, increasing attention has been paid to the decline in quality of life and psychological burden experienced by patients after upper gastrointestinal surgery [15-19]. For these patients, it is crucial not only to detect cancer early and intervene promptly but also to offer sensitive and empathetic communication at the time of diagnosis, addressing the emotional impact of discovering a new malignancy postoperatively.

In the present case, a small, flat, whitish lesion was detected in the non-atrophic mucosa of the lesser curvature at the gastric angle, a region that had previously been part of the native stomach prior to reconstruction. The lesion was more clearly visualized using NBI. Careful biopsy allowed the lesion to remain visible at the time of ESD. Histopathological analysis revealed SRCC limited to the mucosal epithelium, covered by non-neoplastic surface epithelium - a finding consistent with early-phase SRCC. Additionally, by providing thorough explanations and addressing the patient's emotional concerns regarding the discovery of an undifferentiated carcinoma after major surgery, we were able to establish and maintain a strong therapeutic relationship. Such compassionate care should be standard practice for all postoperative patients, especially those who have undergone upper gastrointestinal surgeries, where the psychological and quality-of-life impacts are often profound.

## Conclusions

We reported a rare case of a 4-mm SRCC arising in a reconstructed gastric tube, which was detected during routine follow-up endoscopy and curatively resected with ESD. In this case, the lesion appeared as a flat, discolored area on non-atrophic mucosa and was more clearly visualized with NBI. The lesion was pathologically covered by non-neoplastic epithelium, a feature typical of early-stage SRCC. This case highlights the importance of careful mucosal assessment for subtle lesions that may be easily overlooked during postoperative endoscopic surveillance, even in patients who have likely undergone multiple preoperative endoscopic examinations. It also underscores the importance of providing psychologically supportive communication, as the diagnosis of a new malignancy after major surgery may cause emotional distress. We hope that this case will serve as a useful reference for physicians performing upper gastrointestinal endoscopy in their daily clinical practice.
